# Glucocorticoid-resistant B cell acute lymphoblastic leukemia displays receptor tyrosine kinase activation

**DOI:** 10.1038/s41525-019-0082-y

**Published:** 2019-04-04

**Authors:** Rohit A. Chougule, Kinjal Shah, Sausan A. Moharram, Johan Vallon-Christersson, Julhash U. Kazi

**Affiliations:** 1 Division of Translational Cancer Research, Department of Laboratory Medicine, Lund University, Lund, Sweden; 2 Division of Oncology and Pathology, Department of Clinical Sciences Lund, Lund University, Lund, Sweden

## Abstract

The response of childhood acute lymphoblastic leukemia (ALL) to dexamethasone predicts the long-term remission outcome. To explore the mechanisms of dexamethasone resistance in B cell ALL (B-ALL), we generated dexamethasone-resistant clones by prolonged treatment with dexamethasone. Using RNA-sequencing and high-throughput screening, we found that dexamethasone-resistant cells are dependent on receptor tyrosine kinases. Further analysis with phosphokinase arrays showed that the type III receptor tyrosine kinase FLT3 is constitutively active in resistant cells. Targeted next-generation and Sanger sequencing identified an internal tandem duplication mutation and a point mutation (R845G) in FLT3 in dexamethasone-resistant cells, which were not present in the corresponding sensitive clones. Finally, we showed that resistant cells displayed sensitivity to second-generation FLT3 inhibitors both in vitro and in vivo. Collectively, our data suggest that long-term dexamethasone treatment selects cells with a distinct genetic background, in this case oncogenic FLT3, and therefore therapies targeting FLT3 might be useful for the treatment of relapsed B-ALL patients.

## Introduction

Acute lymphoblastic leukemia (ALL) is one of the most common childhood cancers and can originate both from the B-lineage (B-ALL) and the T-lineage (T-ALL). Glucocorticoids, such as dexamethasone and prednisolone, are important drugs for the treatment of ALL.^1^ In combination with chemotherapeutic agents, glucocorticoids help to achieve clinical remission, and sensitivity to glucocorticoids is considered as a positive prognostic indicator. Patients unresponsive to glucocorticoids often relapse and display poor prognosis. Therefore, understanding the mechanisms behind glucocorticoid insensitivity is important and will help us to develop novel therapeutic modalities. In ALL glucocorticoids induce apoptosis, which is mediated through binding to the glucocorticoid receptor (GR). GR is a nuclear receptor that also acts as a transcription factor. Upon glucocorticoid binding, GR inhibits activator protein-1 (AP-1)- and nuclear factor-κB (NF-κB)-regulated gene transcription, and at the same time promotes glucocorticoid-responsive element-driven gene transcription.^2^ Besides, inhibition of AP-1- and NF-κB-regulated gene transcription, cooperation between AP-1 and GR in transcription,^3^ and crosstalk between NF-κB and GR^4,5^ have been reported, which suggests a context-dependent regulation of AP-1 and NF-κB rather than only inhibitory effects.

Glucocorticoids are useful drugs to induce apoptosis in ALL and have also been widely used to treat inflammatory disorders. However, prolonged use leads to the emergence of glucocorticoid resistance.^6^ The mechanisms of glucocorticoid resistance in leukemia have been studied extensively. Both regulation of expression and function of GR can contribute to glucocorticoid resistance. For instance, activation of NOTCH1 signaling inhibits auto-upregulation of GR expression. Therefore, pharmacological inhibition of NOTCH1 restores glucocorticoid sensitivity.^7^ The relapse-associated mutation in *NR3C1* results in the expression of a non-functional receptor and thereby impairs glucocorticoid sensitivity.^8^ Furthermore, aberrant activation of the PI3K/mTOR pathway has been linked to glucocorticoid resistance in T-ALL.^9^ This is partially mediated by AKT, which phosphorylates GR on S134 and thereby blocks nuclear localization of GR.^10^ Mutations in the transcriptional coactivator CREBBP transcriptionally regulates glucocorticoid-responsive genes, suggesting that functional CREBBP is required for glucocorticoid sensitivity.^11^ Inhibition of glutathione synthesis restored dexamethasone sensitivity in the dexamethasone-resistant B-ALL cell line 697,^12^ suggesting the existence of additional mechanisms of dexamethasone resistance. In this report, we show that cells resistant to dexamethasone harbor activating mutations in the receptor tyrosine kinase FLT3.

## Results

### Prolonged dexamethasone treatment induces dexamethasone resistance in B-ALL cells

**Fig. 1:**
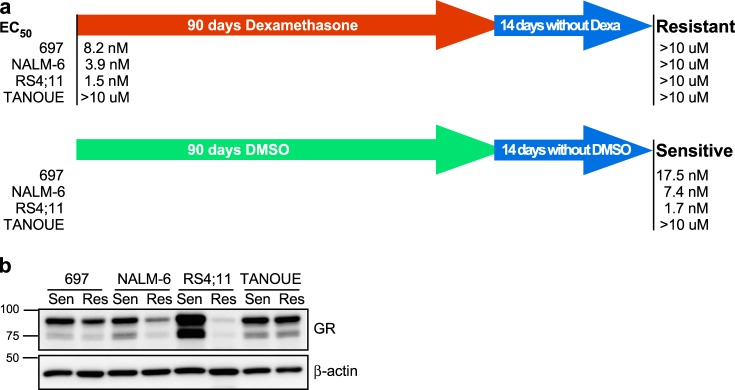
Prolonged dexamethasone treatment induces dexamethasone resistance in B cell acute lymphoblastic leukemia (B-ALL) cells: **a** human B-ALL cell lines 697, NALM-6, RS4;11, and TANOUE were treated with increasing concentrations (from 1 nM to 5 µM) of dexamethasone for 90 days following 14 days of culture in the absence of dexamethasone. Half-maximal effective concentration (EC_50_) was measured using PrestoBlue cell viability assay. Initial EC_50_ values were measured before dexamethasone treatment started. **b** B-ALL cells were lysed and lysates were processed for Western blotting analysis using anti-glucocorticoid receptor (GR) and anti-β-actin antibodies. Blots were derived from the same experiment and were processed in parallel

In order to understand how long-term dexamethasone treatment affects B-ALL cells, we used three dexamethasone-sensitive cell lines: 697 (half-maximal effective concentration (EC_50_) = 8.2 nM), NALM-6 (EC_50_ = 3.9 nM), and RS4;11 (EC_50_ = 1.5 nM), and the dexamethasone-insensitive cell line TANOUE (EC_50_ >10 µM). These cell lines were cultured with an increasing concentration of dexamethasone for 90 days. In parallel, another set of cell lines was cultured with an equivalent amount of dimethyl sulfoxide (DMSO) (which was used to dilute dexamethasone). After 90 days, cells were cultured in normal growth medium for 2 weeks and EC_50_ was measured. We observed that all three dexamethasone-sensitive cell lines cultured in the presence of dexamethasone became highly resistant to dexamethasone, while DMSO-treated cells were still sensitive (Fig. 1a). The relation between dexamethasone sensitivity and GR expression does not always correlate.^13,14^ Therefore, we first checked the GR expression in both dexamethasone-sensitive and -resistant cell lines. The expression of GR remained unchanged in TANOUE cells, while it was reduced in 697 and NALM-6 cells (Fig. 1b). However, while the most sensitive cell line, RS4;11, showed strong GR expression, its expression was completely lost in the corresponding resistant cells (Fig. 1b). These data are in line with previous reports that GR expression is one of the factors relating to dexamethasone sensitivity but not the only factor.^13,14^

### Dexamethasone-resistant RS4;11 cells are sensitive to RTK inhibitors

**Fig. 2:**
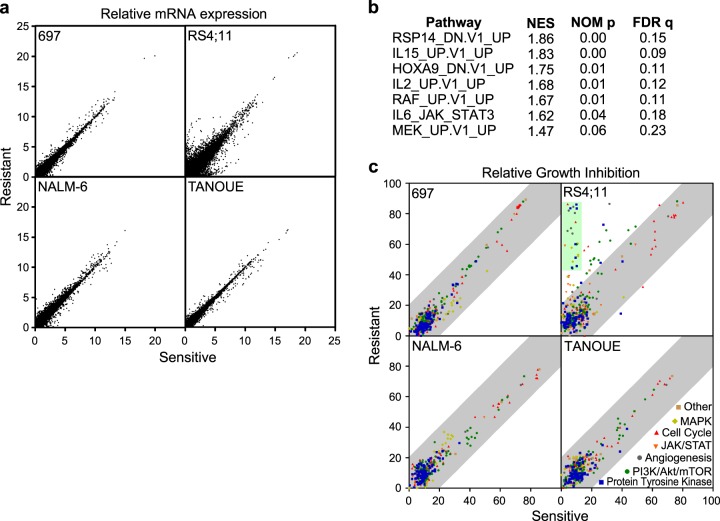
Dexamethasone-resistant RS4;11 cells are sensitive to receptor tyrosine kinase (RTK) inhibitors: **a** Total messenger RNA (mRNA) from both sensitive and resistant B-ALL cell lines were processed for RNA-sequencing (RNAseq). Relative mRNA expression was compared between sensitive and resistant cell lines. **b** Pathway enrichment was determined using Gene Set Enrichment Analysis (GSEA). **c** B-ALL cells were treated with 100 nM of different kinase inhibitors. Relative cell viability was measured by PrestoBlue after 48 h incubation with the inhibitor

To understand the molecular differences between dexamethasone-sensitive and -resistant cell lines, we used RNA-sequencing (RNAseq). We observed that the gene expression patterns were mostly identical between sensitive and resistant lines of 697, NALM-6, and TANOUE cells, whereas RS4;11 cells showed a more scattered expression pattern indicative of differences in gene expression between the two types of cells (Fig. 2a). These data suggest that both dexamethasone-sensitive and -resistant lines of 697, NALM-6, and TANOUE cells keep similar gene expression pattern, while RS4;11-resistant cells show a major difference compared to its parental cell line. As we observed a major variation in gene expression of RS4;11, we checked the pathway enrichment in resistant cells using RNAseq data. We observed enrichment of several kinase and cytokine signaling pathways in resistant RS4;11 cell line (Fig. 2b). Since we observed enrichment of kinase and cytokine signaling pathways in the dexamethasone-resistant RS4;11 cell line, we hypothesized that there is a switch in the dependency of RS4;11 cells from dexamethasone to kinase-related signaling. To identify the possible kinase dependency of RS4;11 cells, we used a panel of 378 inhibitors against different kinases. Both sensitive and resistant lines of NALM-6, 697, and TANOUE cells displayed similar response to the inhibitors, but the resistant RS;411 cell line displayed increased sensitivity to several receptor tyrosine kinase (RTK) inhibitors compared to the corresponding sensitive cell line (Fig. 2c). Taken together, these data suggest that the mechanism behind the resistant phenotype of RS4;11 is different from that of NALM-6, 697, and TANOUE cell lines.

### Dexamethasone-resistant RS4;11 cells display tyrosine phosphorylation of FLT3

**Fig. 3:**
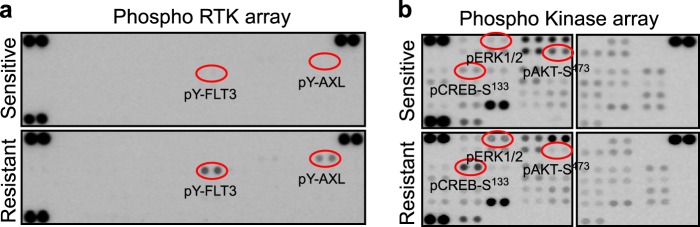
Dexamethasone-resistant RS4;11 cells display tyrosine phosphorylation of FLT3: **a** Lysates from RS4;11 cells were used to incubate phospho-receptor tyrosine kinase (RTK) array membranes and then processed following manufacturer’s instructions. **b** Phospho-kinase arrays were incubated with lysates from RS4;11 cells and then processed following the manufacturer’s instruction

Since we did not observe any major differences between the gene expression and kinase inhibitor response, we suggest that the resistance of 697 and NALM-6 is probably mediated by reduced expression of GR or due to a loss-of-function mutation in GR. Several other mechanisms have also been described and discussed in the Introduction section.^7–12^ However, the difference in gene expression in the two RS4;11 cell lines and their differential response to kinase inhibitors evoked our interest. Coinciding with the development of a resistant phenotype, the RS4;11 cells completely lost GR expression. Most likely this is due to the fact that a small fraction of cells that initially were lacking GR expression were selected for during the long-term exposure to dexamethasone, and that selected for cells that carry different genetic mutations. Since we observed that dexamethasone-resistant RS4;11 cells are sensitive to several RTK inhibitors, we checked for activation of RTKs in this cell line using a human proteome phospho-RTK array. Surprisingly, we observed strong tyrosine phosphorylation of FLT3 and weak tyrosine phosphorylation of AXL in resistant cells, which could not be seen in sensitive cells (Fig. 3a). Furthermore, using a phosphokinase array we observed that phosphorylation of ERK1/2 and of CREB at S133 was enhanced in resistant cells (Fig. 3b). Collectively, our data suggest that dexamethasone-resistant RS4;11 cells display dependency of constitutively active RTK signaling.

### Dexamethasone-resistant RS4;11 cells carry oncogenic mutants of FLT3 and respond to FLT3 inhibition

**Fig. 4:**
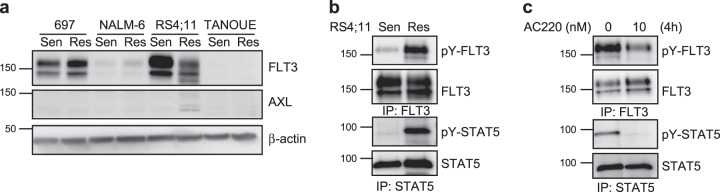
Dexamethasone-resistant RS4;11 cells carry oncogenic FLT3 and respond to FLT3 inhibitor: **a** B-ALL cells were lysed and lysates were processed for Western blotting using anti-FLT3 (blot from Fig. 1b was used to probe), anti-AXL, and anti-β-actin antibodies. **b** RS4;11 cells were lysed and immunoprecipitated with either anti-FLT3 or anti-STAT5 antibodies and then processed for Western blotting using anti-phosphotyrosine (4G10), anti-FLT3, and anti-STAT5 antibodies. **c** Dexamethasone-resistant RS4;11 cells were treated with 10 nM AC220 for 4 h before lysis. Lysates were immunoprecipitated with either anti-FLT3 or anti-STAT5 antibodies and then processed for Western blotting using phosphotyrosine (4G10), anti-FLT3, and anti-STAT5 antibodies. Blots were derived from the same experiment and were processed in parallel

We then checked the expression of FLT3 and AXL in RS4;11 cell lines. We observed strong expression of FLT3 in dexamethasone-sensitive RS4;11, where the fully glycosylated, mature FLT3 band was stronger than the partially glycosylated, immature band (Fig. 4a), which is a characteristic of cells expressing wild-type FLT3. The observation that the partially glycosylated, immature FLT3 band was stronger in dexamethasone-resistant RS4;11 cells (Fig. 4a) raised the possibility that the resistant cells carry an oncogenic internal tandem duplication (ITD) mutation in FLT3, which typically gives this pattern of expression.^15,16^ This is also supported by the fact that resistant RS4;11 cells showed constitutive tyrosine phosphorylation of FLT3 as well as constitutive STAT5 phosphorylation (Fig. 4b) and that the second-generation FLT3 inhibitor AC220 could block tyrosine phosphorylation of both FLT3 and STAT5 (Fig. 4c).

### Dexamethasone-resistant RS4;11 cells carry FLT3-ITD and FLT3-R845G mutations

**Fig. 5:**
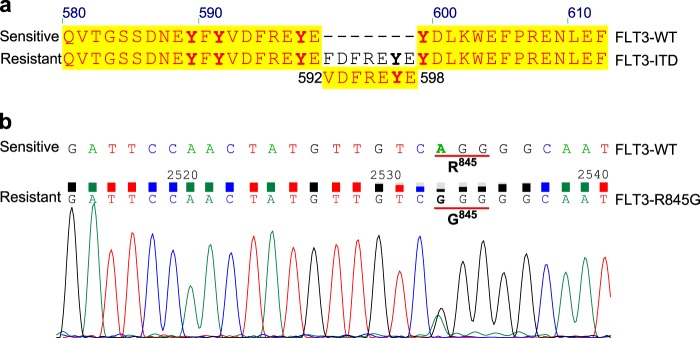
Dexamethasone-resistant RS4;11 cells carry FLT3-ITD (internal tandem duplication) and FLT3-R845G mutations: **a** Sequence alignment of data for wild-type and mutant FLT3. **b** Sanger sequencing data for the R845G mutant of FLT3

To verify the presence of FLT3 mutations and also in order to see whether any other oncogenic mutations exist in RS4;11 cells, we used targeted sequencing of 600 cancer-related genes. We identified a FLT3 point mutation (c.2533A>G, R845G, ratio 65%, coverage 1504×) and an FLT3-ITD mutation (p.E598_Y599insFDFREYE 22%, coverage 487×) (Fig. 5a). The point mutation was further confirmed by Sanger sequencing (Fig. 5b). FLT3-ITD is a well-studied oncogenic mutation and R845G has also been shown to be a constitutively activating mutation.^17^

### Dexamethasone-resistant RS4;11 cells are sensitive to the second-generation FLT3 inhibitors AC220 and crenolanib

**Fig. 6:**
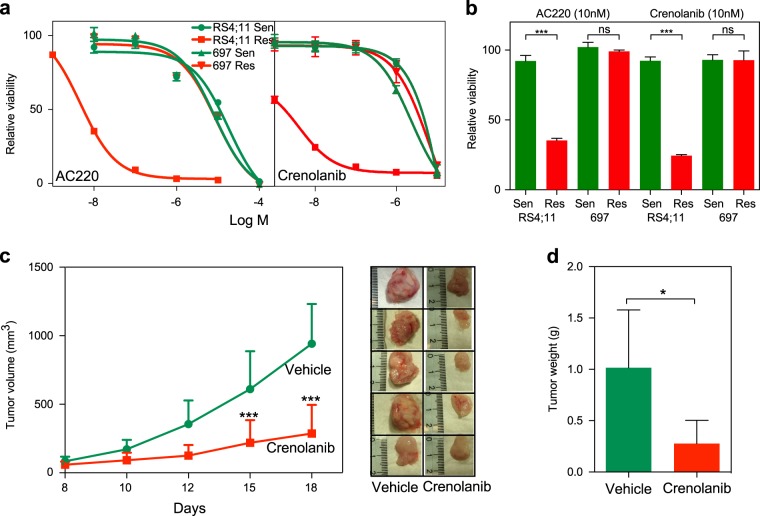
Dexamethasone-resistant RS4;11 cells are sensitive to the second-generation FLT3 inhibitors AC220 and crenolanib: **a** RS4;11 and 697 cells were treated with different concentration of AC220 and crenolanib for 48 h. Cell viability was measured by PrestoBlue. **b** RS4;11 and 697 cells were treated with 10 nM of AC220 or crenolanib for 48 h. Cell viability was measured by PrestoBlue. One-way analysis of variance (ANOVA) with Bonferroni post test was used for statistical analysis. ns, not significant; ***p < 0.001. **c** Two million dexamethasone-resistant RS4;11 cells were injected subcutaneously into 10 NSG mice. One week after injection, half of them were treated with the vehicle and half of them were treated with 12 mg/kg crenolanib one every second day from 8th to 16th day (intraperitoneally). Tumor volume was measured on days 8, 10, 12, 15, and 18 after treatment. One-way ANOVA with Bonferroni post test was used for statistical analysis. ***p < 0.001. **d** Tumor weight was measured after sacrificing mice on the 18th day after treatment. Student’s *t* test was used for statistical analysis. *p < 0.05

Since dexamethasone-resistant RS4;11 cells harbor oncogenic mutations in FLT3, we have tested the possibility of using the second-generation FLT3 inhibitors AC220 and crenolanib. Both the inhibitors significantly reduced the growth of dexamethasone-resistant RS4;11 cells, while the growth of dexamethasone-sensitive RS4;11 and 697 cells or dexamethasone-resistant 697 cells remained unchanged (Fig. 6a, b). Furthermore, in a mouse xenograft model, crenolanib delayed tumor formation of dexamethasone-resistant RS4;11 cells (Fig. 6c, d). Taken together, data suggest that dexamethasone-resistant RS4;11 cells are dependent on the activity of oncogenic FLT3 signaling.

## Discussion

In this study, we used three dexamethasone-sensitive B-ALL cell lines from three different genetic backgrounds to generate dexamethasone-resistant cell lines. Although the 697 cell line carries an *E2A-PBX1* (*TCF3-PBX1*) fusion, RS4;11 carry an *MLL-AF4* fusion and NALM-6 carry an *NRAS* mutation, all three cell lines displayed similar sensitivity to dexamethasone (EC_50_ <10 nM). E2A-PBX1 fusion acts as a constitutively active transcription factor that downregulates the expression of *CDKN2A*.^18^ *CDKN2A* encodes two distinct proteins (p16^INK4A^ and p14^ARF^), which are well-known regulators of the cell cycle. E2A-PBX1 does not act as a transcriptional repressor, but this fusion protein enhances expression of *BMI-1*,^18^ which is known to be a lymphoid oncogene and functions as a transcriptional repressor. On the other hand, MLL-AF4 suppresses the expression of another cell cycle regulatory protein, p27^KIP1^ through direct transcriptional repression of *CDKN1B*.^19^ Furthermore, ALL patients with *MLL-AF4* rearrangements overexpress *HOXA9*,^20^ which is a transcription factor that has been shown to be important for the proliferation and survival of *MLL*-rearranged leukemias.^21^ HOXA9 mediates upregulation *BCL2* expression, which in turn provides survival signals to the leukemic cells.^22^ Therefore, combinatorial use of dexamethasone and BCL2 inhibitor displayed a synergistic effect in inhibition of leukemia.^23^ Although patients with *MLL-AF4* fusion respond to glucocorticoid-based chemotherapy, this group of patients are considered to have a poor prognosis and have about 60% disease-free survival.^24^ Current studies suggest that this group of patients shows resistance to glucocorticoids, which has been shown to be at least partially mediated by constitutive activation of mitogen-activated protein kinase signaling.^25–27^ Here we provide evidence that cells that are resistant to dexamethasone display constitutive activation of RTK signaling.

All three dexamethasone-sensitive B-ALL cell lines became resistant during 90 days treatment of dexamethasone, suggesting that prolonged treatment-induced resistance to dexamethasone in vitro. This was independent of the GR expression levels as dexamethasone-resistant 697 cells also express a similar level of GR as sensitive cells. However, while both 697 and NALM-6 cells kept a certain level of GR expression after 90 days of dexamethasone treatment, expression was almost lost in RS4;11 cells. Besides that, RS4;11 cells displayed a major deviation with respect to gene expression and kinase inhibitor sensitivity when comparing the dexamethasone-sensitive and -resistant cells, suggesting that this cell line harbors a different mechanism of dexamethasone resistance than the other two cell lines. Furthermore, RS4;11 cells resistant to dexamethasone showed constitutive activation of FLT3.

A relationship between *MLL* rearrangement and FLT3 expression has been established in several studies. For example, FLT3 expression was found to be consistently higher in ALL patients positive for *MLL* rearrangement,^28^ and mutations in the activation loop of FLT3 that confer constitutive activation of FLT3 was identified in 17% of *MLL*-rearranged ALL patients.^29^ However, higher FLT3 expression and oncogenic mutations are not exclusive to *MLL*-rearranged ALL, while it occurs frequently in hyperdiploid ALL and less frequently in *TEL-AML1* fusion ALL.^30,31^ We observed that the 697 and RS4;11 cell lines express higher levels of FLT3, while its expression was undetectable in TANOUE cells and low in NALM-6 cells. Since dexamethasone-sensitive RS4;11 cells do not have any FLT3 activation, and since it is unlikely that dexamethasone will induce mutation in *FLT3*, it seems that a small fraction of RS4;11 cells carry FLT3 mutations from the beginning. Dexamethasone selection probably selects for cells that are dependent on FLT3 signaling.

Collectively, our data suggest that dexamethasone-resistant RS4;11 cells are a subpopulation of B-ALL cells that carry FLT3-ITD or R845G mutations, and therefore it could prove useful to screen B-ALL patients who are resistant to dexamethasone for mutations in FLT3, which then could be targeted with FLT3 inhibitors that are already available on the market.

## Methods

### Antibodies and chemicals

Anti-GR (sc-8992; 1:1000 dilution), anti-β-actin (sc-47778; 1:1000 dilution), anti-AXL (sc-1096; 1:1000 dilution), anti-FLT3 (sc-479; 1:1000 dilution), and anti-STAT5 (sc-835; 1:1000 dilution) were from Santa Cruz Biotechnology (Dallas, TX, USA). Anti-phospho-tyrosine antibody 4G10 (05-321; 1:1000 dilution) was from Millipore. Dexamethasone (D4902, Sigma-Aldrich, St. Louis, MI, USA) was dissolved in DMSO. All uncropped blots are available in supplementary figure.

### Cell culture and generation of dexamethasone-resistant B-ALL cell lines

The B-ALL cell lines RS4;11, 697, NALM-6, and TANOUE were purchased from the DSMZ (Braunschweig, Germany). B-ALL cell lines were cultured in RPMI-1640 medium supplemented with 10% heat-inactivated fetal bovine serum (FBS), 100U/ml penicillin and 100 µg/ml streptomycin. All B-ALL cell lines were treated with dexamethasone and doses were doubled when the treated cells started to proliferate at an equal rate to the untreated parental cells. The doses were increased at regular intervals until 5 µM concentration was reached. The resistant cells were further grown for 2 weeks in the absence of inhibitors.

### Drug sensitivity assay

Sensitive and resistant B-ALL cells were grown in RPMI-1640 medium supplemented with 10% FBS, 100U/ml penicillin, and 100 μg/ml streptomycin. Cells were then seeded in 96-well plates (10,000 cells per well) in the presence of different concentrations of dexamethasone. After 48 h, 10 μl of PrestoBlue was added to each well, followed by 2 h of incubation. Cell viability was calculated according to the manufacturer’s protocol. A kinase inhibitor library including 378 kinase inhibitors was obtained from Selleck Chemicals (Houston, TX, USA). Stock solutions of 10 mM inhibitor were diluted to 100 nM using the cell culture medium. Cell viability assays using PrestoBlue were used to examine the effect of inhibitors.

### Phosphokinase arrays

Proteome Profiler Human Phospho-RTK Array Kit **(**ARY001B) and Proteome Profiler Human Phospho-Kinase Array Kit (ARY003B) were obtained from R&D System (Minneapolis, MN, USA). Dexamethasone-sensitive and -resistant cells were lysed and the lysates were processed according to the manufacturer’s protocols.

### Targeted sequencing of cancer panel

INVIEW Oncopanel All-in-one service from Eurofins Genomics provided analysis of 597 key cancer-specific genes. Total genomic DNA from dexamethasone-sensitive and -resistant cells was purified using Qiagen DNeasy Blood and Tissue Kit (69504), and then sent to Eurofins Genomics for processing.

### Mouse xenograft studies

Ten female non-obese diabetic/severe combined immunodeficiency gamma (NSG) mice (each weighing ~20 g and housed by the Laboratory Animal Facilities at Medicon Village, Lund University) were injected with 2,000,000 cells with 1:1 Matrigel subcutaneously. Mice were then divided into two groups and treated either with crenolanib or with the vehicle. One week after injection of cells, mice were treated alternative days by intravenous injection of 12 mg/kg crenolanib or vehicle for additional days until the tumor reached a size of 1 cm^3^ (8th to 16th day). Drug efficacy was checked by monitoring the tumor growth in both groups and by regularly measuring the body weight and tumor volume of the mice. Mice were sacrificed after the size of the tumors had reached about 1 cm^3^.

### Ethical consideration

Mice were maintained by following regulation approved by the Lund University. All animal experiments were performed under an ethical permit from the Swedish Animal Welfare Authority.

### RNAseq analysis

The RNA quality was analyzed using a Bioanalyzer (Agilent) and samples with an RNA integrity greater than seven were further analyzed. Subsequently, RNAseq was performed using TruSeq Stranded mRNA Kit for NeoPrep from Illumina and the sequencing was performed using Illumina NextSeq 500 instrument. RNAseq data analysis was performed using a pipeline where Demultiplexing step involves in reorganizing the FASTQ files based on the sample index information, and generating the statistics and reporting files, which was performed using the bcl2fastq2 software (Illumina), Masking step involves filtering ribosomal RNA (GenBank loci NR_023363.1, NR_003285.2, NR_003286.2, NR_003287.2, X12811.1, U13369.1), PhiX (phiX174), and Illumina control (NC_001422.1), and repeat sequence analysis was performed using the bowtie2 program. Mapping steps, after filtration of remaining reads, were aligned to the human genome reference (UCSC hg38 build), performed using TopHat2 program. Expression count step, expression levels, and fragments per kilobase of exon per million mapped reads were calculated using Cufflinks 2.2.1 program.

### Statistical analysis

Statistical analysis was performed using GraphPad Prism 5.0. One-way analysis of variance or Student’s *t* test was used where needed.

### Reporting Summary

Further information on research design is available in the Nature Research Reporting Summary.

## Supplementary information

Uncropped images of all blots

Reporting summary

## Data Availability

All raw data are available upon request. RNAseq data are available at the ArrayExpress (E-MTAB-7781). Raw data supporting FLT3 mutations in RS4;11 cells have been deposited to figshare.com 10.6084/m9.figshare.7828172.

## Supplementary information

**Supplementary information** accompanies the paper on the *npj Genomic Medicine* website (10.1038/s41525-019-0082-y).
