# Supplementary figures and images for: Glucocorticoid-resistant B cell acute lymphoblastic leukemia displays receptor tyrosine kinase activation
Source: NPJ Genom Med. 2019 Apr 4;4:7. doi: 10.1038/s41525-019-0082-y (PMC6449402; doi:10.1038/s41525-019-0082-y)

Figure 1B

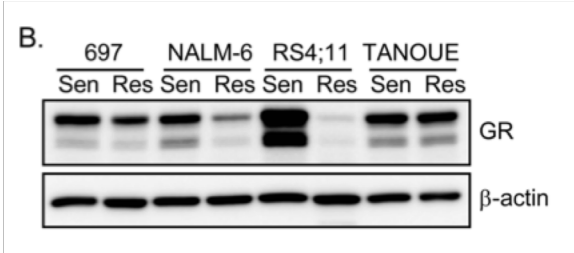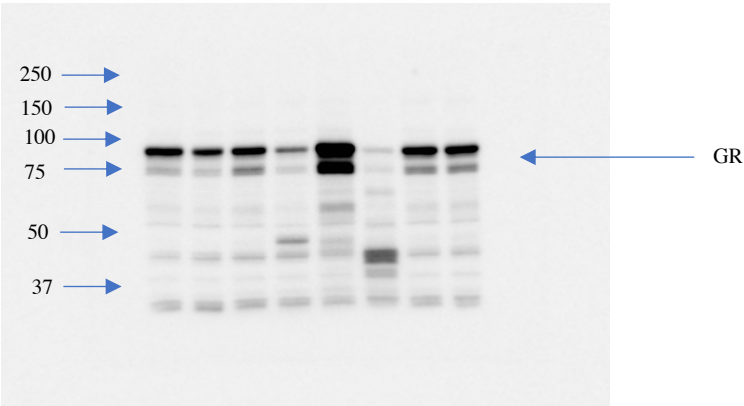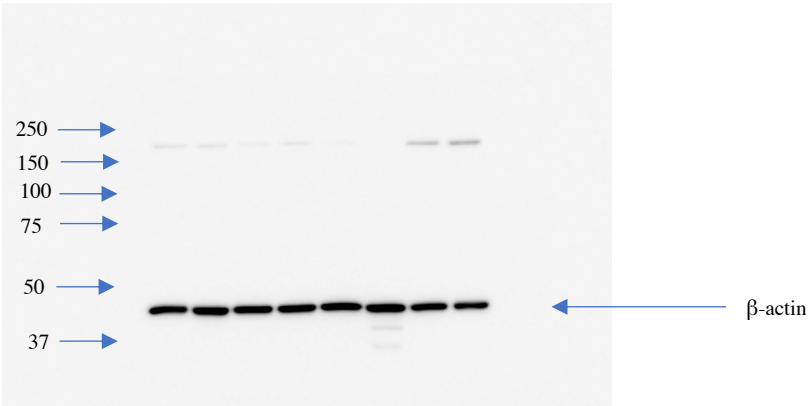

Figure 4A

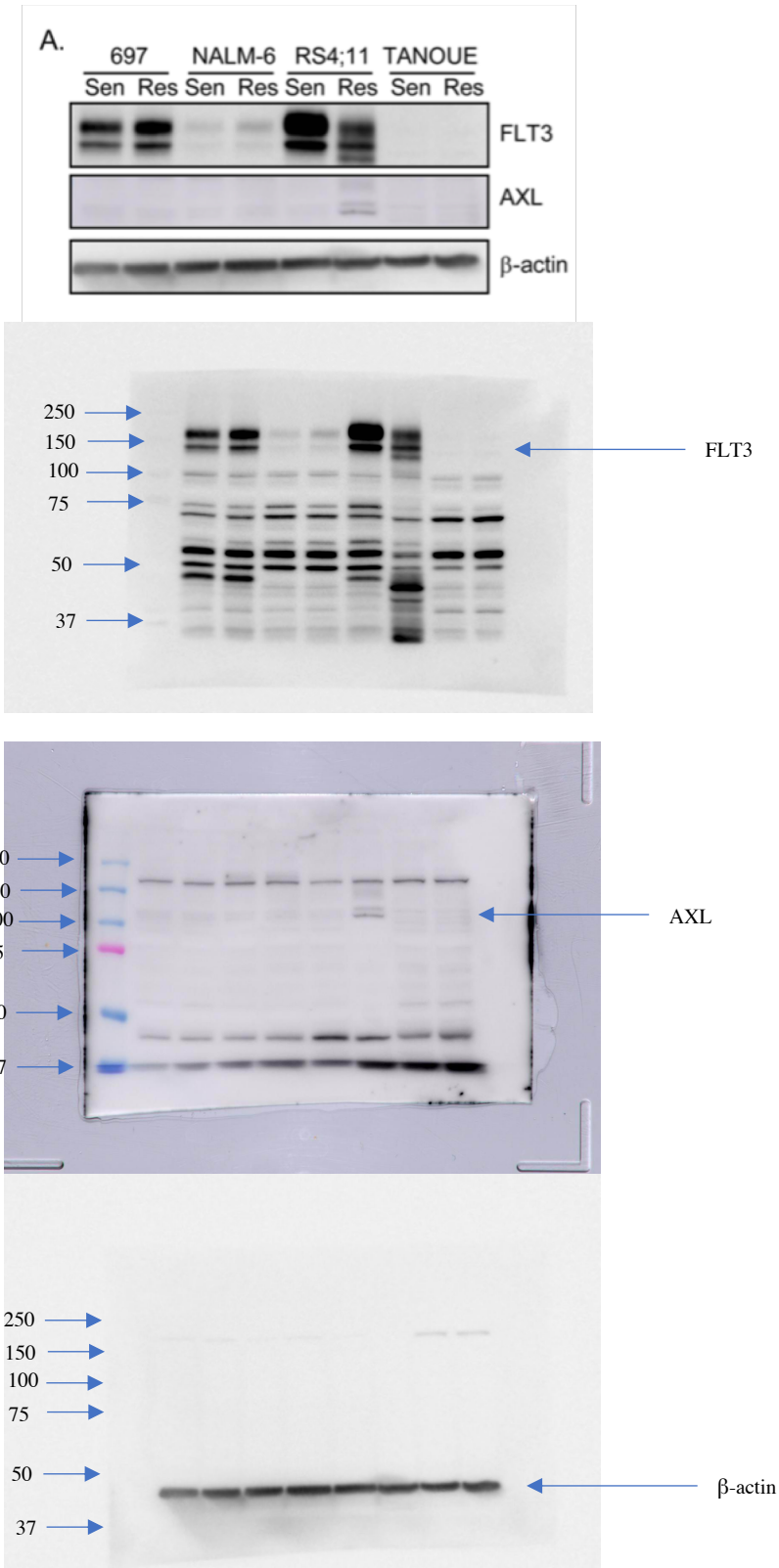

Figure 4B

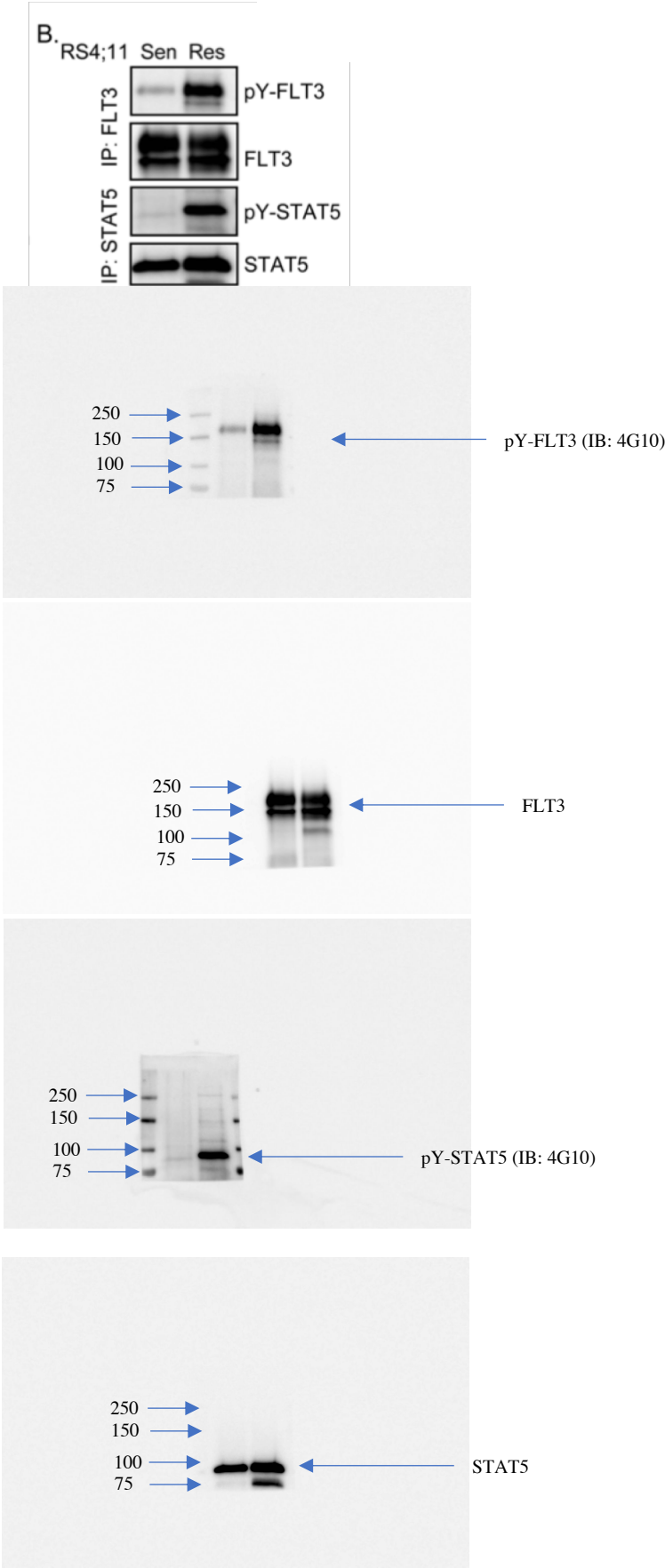

Figure 4C

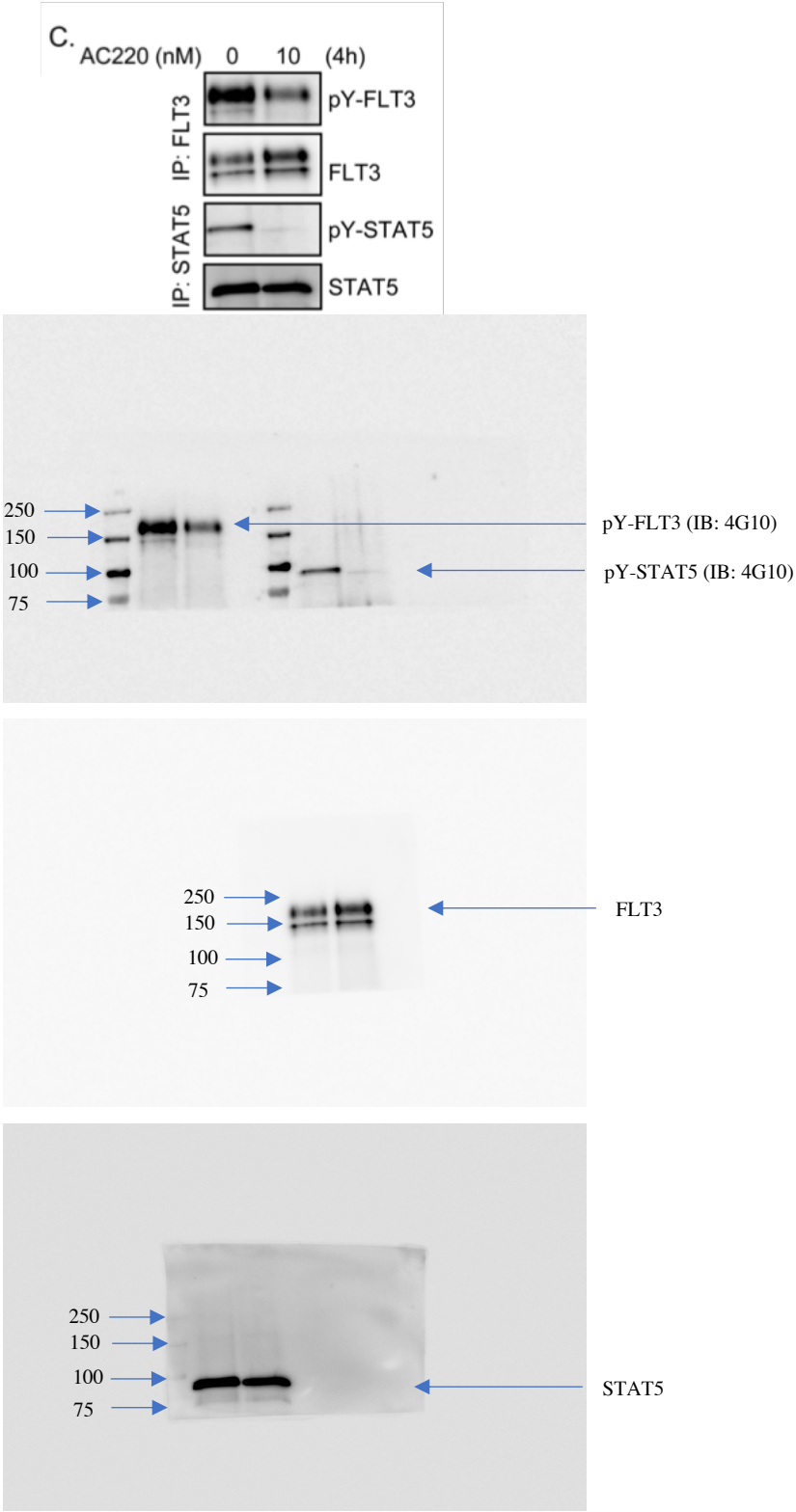

Supplement: Supplementary file 1 — Uncropped images of all blots [file 41525_2019_82_MOESM1_ESM.pdf]
